# The effect of breaches of the psychological contract on the job satisfaction and wellbeing of doctors in Ireland: a quantitative study

**DOI:** 10.1186/s12960-020-00534-3

**Published:** 2020-11-12

**Authors:** Aedin Collins, Alexandra Beauregard

**Affiliations:** Birkbeck, University of London, Malet St, Bloomsbury, London, WC1E 7HX UK

**Keywords:** Job satisfaction, Wellbeing, Psychological contract, Non-consultant doctors

## Abstract

**Background:**

Medicine is one of the most popular college degrees at both undergraduate and postgraduate level. Despite this, morale and wellbeing in doctors at all levels internationally is reportedly low. Long hours and stressful working environments have been implicated as the cause of this. The psychological contract is the implicit expectations and mutual obligations held between an employee and employer. Breaches in this contract can lead to strong negative emotional responses. This study will examine the psychological contract of non-consultant doctors and gain further insight into their job satisfaction and wellbeing. It aims to ascertain the effect of breaches of the psychological contract on their job satisfaction and wellbeing.

**Methods:**

This is a quantitative study performed using a questionnaire on a closed online forum. Job satisfaction, wellbeing and breaches of the psychological contract were measured using pre-existing and pre-validated scales. Statistical analysis was performed to determine the effect of breaches of the psychological contract on job satisfaction and wellbeing.

**Results:**

This study ascertained that training and career development were the most important areas of the psychological contract for non-consultant doctors and training and organizational support the most important breaches. It found, overall, positive levels of job satisfaction and wellbeing. A statistically significant relationship between breaches of the psychological contract and job satisfaction and wellbeing was found.

**Conclusion:**

This study provides an insight into the psychological contract of non-consultant doctors in Ireland. By doing so, it identifies areas for change which may improve their future job satisfaction and wellbeing.

## Introduction

The delivery of healthcare is constantly changing and evolving, as are the pressures being placed on medical professionals. Within recent years, there has been increasing concern regarding a decline in doctors’ wellbeing [1–3]. Reported levels of burnout and stress and psychological distress are high [2, 4]. This has manifested in difficulties with recruitment and retainment of appropriately trained staff, rota gaps and reported lack of continuity of care for patients [3, 5, 6]. In the late 1990s and early 2000s  it was hypothesized that changes in job roles, increased medicolegal cases and lack of autonomy were leading to reduced job satisfaction among doctors [7]. Working in an overstretched and under-resourced health system may also lead to the demands of the job outweighing job control and resources, leading to a further reduction in job satisfaction [8].

Postgraduate medical training in Ireland has also gone through significant changes in the last 20 years. The Buttimer report in 2006 identified the need to provide attractive training facilities with high-quality schemes to result in fully qualified doctors able to deliver the health needs of the population of Ireland [9]. This resulted in the formalization of training agreements with both hospitals and postgraduate training bodies. However, there is no clear career path after the completion of training, with consultant posts not always available at this stage. Ireland currently has the lowest number of consultants per capita in the EU [10]. In addition, Ireland has been amongst the slowest countries in implementing the European Working Time Directive [11]. As a result of that, many Irish doctors still do 24-hour on-call shifts and work in a classic team structure—consultant, registrar and senior house office. This is in contrast with, for example, the NHS, where most junior doctors work in shift patterns. There is a significant issue with retention of doctors in Ireland, the normalization of extreme working hours identified as a key driver of doctor emigration and a key deterrent of repatriation [12, 13] has been implicated as a cause for significant issues with retention of doctors in Ireland [14].

Of doctors who remain in Ireland, over one-third have reported some degree of psychological distress, with depression being reported in 7.1%, anxiety in 6.1% and stress in 9.5% [15]. These levels are significantly higher in non-consultant doctors than in consultants and are 2.5 times that of the general population [15]. The term non-consultant doctors incorporates both doctors in official training schemes and those working as senior house officers, registrars or fellows in stand-alone posts.

Ireland has been in a period of austerity since 2008. From 2009 to 2013, funding to the Health Service fell by 3.3 billion euros and staffing levels fell by 10% [8]. This has resulted in a health system under pressure, with reduced bed numbers and rising waiting list times [8]. Non-consultant doctors in Ireland have practised mainly within this time of austerity and within a health system struggling to meet the needs of its population, with significant reductions in public sector salaries over this time [12].

This study will use psychological contract theory as a lens through which to examine determinants of job satisfaction and wellbeing among non-consultant doctors in Ireland. The psychological contract is a widely studied construct within the organizational psychology literature and refers to the beliefs and expectations held by both employee and employer regarding their mutual obligations within a workplace [16]. Historically, there have been two main forms of psychological contract. Relational contracts are present in long-term employment and associated with employee loyalty and job security, a sense of commitment to an organization and a feeling that short-term sacrifices such as working overtime will be rewarded in the longer term [17]. Mutual investment by both employee and employer is at the core of a relational contract [16]. In contrast, transactional contracts are considered more common in short-term employment or roles with specific, defined duties, e.g., if an employee works a certain number of hours, they expect a certain amount of pay. An employee with a transactional contract is less likely to work outside their job role, they find the prospect of finding work elsewhere less concerning and they are more likely to move organizations frequently [16, 17]. A third form of psychological contract, known as the hybrid or balanced contract, combines the time frame and mutual rewards of a relational contract with the specified demands of a transactional one [16].

In one previous study on the psychological contract of doctors, Bunderson described how contracts might change from relational to transactional [18]. Recent research suggests that doctors may no longer view their career as simply a vocation, but instead have expectations of an acceptable work–life balance and appropriate financial rewards for the level of effort expended [12]. This may be leading to a change from a historical relational contract among doctors to, if not a transactional contract, at least a hybrid one. Similarly, while doctors in non-consultant positions and training may have a relational contract with the overall health system in which they plan to work and develop their careers, they move hospitals and departments very regularly—every two to three months in some cases—so within their current workplace they may have a transactional contract [18].

When one party in the employment relationship fails to fulfil their obligations toward the other, breach is said to occur. Breaches in the psychological contract that have a severe negative emotional effect on the employee are known as violations [19]. In professions other than medicine, psychological contract breach has been associated with decreased work commitment, engagement, trust, job satisfaction and emotional wellbeing among employees [20]. Breaches are more commonly seen in the public sector and large organizations [21], but there has to date been very little research on the psychological contract and the potential implications of breach among doctors.

This study has two aims. First, to gain insight into the levels of job satisfaction and wellbeing among non-consultant doctors in Ireland. Second, to assess the importance of specific elements of the psychological contract to non-consultant doctors in Ireland, to identify the most common breaches and violations, and to examine the effect of psychological contract breach and violation on doctors’ reported job satisfaction and wellbeing.

## Methods

### Sample and procedure

An online survey was shared on a closed Facebook forum for non-consultant doctors in Ireland, of which the author was a member, in the second quarter of 2019. The forum is mainly used for accommodation swaps around times of rotations and other administrative matters. All members are verified by administrators. Ethical approval for the study was granted by the author’s institution and informed consent for participation in the study was sought electronically prior to commencement of the survey. No identifiable information was collected and all data were stored securely.

### Measures

Job satisfaction was measured using the Aggregate Job Satisfaction Scale [22], a 13-item questionnaire with a six-point Likert response scale. It examines different areas of job satisfaction from work, salary, promotion to teamwork and interrelationships with colleagues. A sample question is, “Please indicate how dissatisfied/satisfied you are with the promotion opportunities you have.” Due to the specificity of the cohort being studied, some changes were made to the wording of the items, e.g., “company” was replaced by “hospital/clinic”.

Wellbeing was measured with the Scale of Positive and Negative Experiences (SPANE) and the Flourishing Scale [23]. SPANE looks at how much time one has spent feeling six positive (e.g., happy, pleasant) and six negative (e.g., unhappy, unpleasant) emotions within the previous 4 weeks [23]. Each emotion is scored on a five-point scale from 1 = very rarely to 5 = very often and aggregated to form a positive feeling score and a negative feeling score. An overall score known as the ‘affect balance’ is reached by subtracting the negative feeling score from the positive feeling score. The result can vary from − 24 (the most negative) to + 24 (the most positive).

The Flourishing Scale is an indicator of overall psychological wellbeing and contains eight statements describing day-to-day functioning including relationships with peers, feelings of purposefulness, etc., which are measured on a seven-point Likert response scale ranging from strong disagreement to strong agreement. A sample item is, “I actively contribute to the happiness and well-being of others”.

Psychological contract breach was assessed with Turnley and Feldman’s (1999) 16-item measure. Respondents scored the importance to them of various aspects within the psychological contract (e.g., training, career development, job security) from 1 to 10. The same items were then scored again according to the amount of each item received from the respondent’s employer compared to how much respondents were led to believe they could expect. Scores ranged from − 2 (much less than promised) to + 2 (much more than promised). Responses were reverse scored so the higher the score, the more significant the breach. The degree of emotional response to the breach, or the degree of violation, was measured by multiplying degree of breach by the importance of each aspect, and then adding this score for all elements together to give a total degree of violation of the psychological contract as a whole [24].

### Analysis

Descriptive statistics were calculated using SPSS software. To determine the relationship between breaches in the psychological contract and job satisfaction and wellbeing, linear regression analysis was conducted. Significance was predetermined at *p* < 0.05.

## Results

There were 340 respondents, 281 of whom provided complete responses to the main body of the questionnaire. There were 277 respondents to the demographic section. The majority of participants (75%) were women and nearly 80% of participants were under 35 years old. The vast majority of respondents (88%) had graduated within the past 9 years, with 44% having graduated within the past 4 years and another 44% having graduated between 5 and 9 years ago. Senior house officers accounted for 32.5% and specialist registrar for 42.5%. The remaining respondents were interns, registrars or post-certificate of completion of speciality training (CCST) fellows.

### Job satisfaction

Scores for items in the Job Satisfaction Scale are displayed in Table 1. Composite job satisfaction scores have a potential range of 13–78. Results from this cohort ranged from 19 to 74 with a mean of 48 (SD of 11.52), indicating an overall positive level of job satisfaction among non-consultant doctors. For individual items, satisfaction with personal relationships with co-workers scored highest with a mean score of 4.95 (SD 1.04) out of a maximum score of 6. Other high-scoring elements included the ‘work you do’ with a mean of 4.34 (SD 1.33), functioning of work team with a mean of 4.28 (SD 1.25) and coordination of the team with a mean of 4.23 (SD 1.23). Human resource management and hospital/practice management scored the lowest of the 14 domains with means of 2.52 (SD 1.5) and 2.61 (SD 1.36), respectively.

**Table 1:** Demographics

	%	*n*
Gender		
Male	23.8	66
Female	75.45	209
Preferred not to answer	0.72	2
Age		
18–24	2.53	7
25–34	78.34	217
35–44	18.05	50
> 45	1.08	3
Years post-graduation		
0–4	44.04	122
5–9	43.68	121
> 10	12.27	34
Studied medicine as		
Undergraduate	69.31	192
Postgraduate	30.69	85
Career stage		
Intern	5.42	15
Senior house officer	32.49	90
Registrar	21.66	60
Specialist registrar	33.21	92
Fellow	7.22	20

### Wellbeing

Scores for items in the Flourishing Scale can be seen in Table 2. The highest scoring item was ‘being a good person and living a good life’ with a mean score of 5.59 out of 6 (SD 1.11) and the lowest was ‘people respect me’ with a mean of 4.97 (SD 1.37). The mean individual total score was 42.14 with a standard deviation of 8.84, with scores ranging from 12 to the maximum score of 56.

**Table 2:** Job Satisfaction Scale

	Minimum	Maximum	Mean	SD	Variance
The personal relationships with your co-workers	1	6	4.94	1.05	1.1
The work you do	1	6	4.36	1.33	1.77
The functioning of your work team	1	6	4.28	1.25	1.55
The coordination among members of your work team	1	6	4.23	1.26	1.59
The opportunities to participate in the decisions that affect your work team	1	6	4	1.35	1.84
The hospital/practice, considered overall	1	6	3.77	1.34	1.79
The promotion opportunities you have	1	6	3.67	1.47	2.17
The salary you get	1	6	3.65	1.38	1.91
The direct supervision you receive	1	6	3.6	1.57	2.45
The physical working conditions you have (light, temperature, noise, etc.)	1	6	3.27	1.54	2.37
The training opportunities provided by your hospital/practice	1	6	3.21	1.49	2.23
The hospital/practice management	1	6	2.61	1.36	1.85
The human resources management in your hospital/practice	1	6	2.52	1.5	2.24

In the Scale of Positive and Negative Experiences, the most commonly reported feelings were ‘good’ and ‘pleasant’ with means of 3.53 (SD 0.8) and 3.56 (SD 0.79), respectively. ‘Unpleasant’ received the lowest score with a mean of 2.9 (SD 0.87).

By subtracting the result of the scale of negative experiences from the results of the scale of positive experiences, the affect balance was generated. A negative score shows more time being spent with negative emotions and a positive score shows more time being spent with positive emotions and the potential range is from − 24 to + 24. The responses to this questionnaire ranged from − 23 to + 21, with a mean of 2.4 (SD 7.82), indicating more time spent feeling positive rather than negative emotions.

### Psychological contract

Scores for importance and fulfilment of items in the Psychological Contract scale can be seen in Table 3. The most important item identified by non-consultant doctors was training with a mean score of 9.26 (SD 1.04). Other important areas identified were career development with a mean score of 8.79 (SD 1.31) and advancement with a mean score of 8.65 (SD 1.36) (Table 4).

**Table 3:** Flourishing Scale

	Minimum	Maximum	Mean	SD
I am a good person and live a good life	1	7	5.59	1.11
I am competent and capable in the activities that are important to me	1	7	5.48	1.19
My social relationships are supportive and rewarding	1	7	5.44	1.5
I actively contribute to the happiness and well-being of others	1	7	5.37	1.29
I am engaged and interested in my daily activities	1	7	5.09	1.5
I am optimistic about my future	1	7	5.03	1.59
People respect me	1	7	4.97	1.37

**Table 4:** Importance and fulfillment of aspects of the psychological contract

	Importance		Fulfillment	
	Mean	SD	Mean	SD
Training	9.26	1.04	2.09	1.02
Career development	8.79	1.31	2.48	0.94
Advancement	8.65	1.36	2.58	0.93
Organizational support	8.64	1.44	1.98	0.96
Supervisory support	8.48	1.47	2.47	1.07
Job security	8.39	1.52	2.64	0.97
Feedback	8.26	1.45	2.24	0.94
Salary	8.15	1.63	2.47	0.84
Decision-making input	8.12	1.37	2.88	1
Bonuses/overtime payments	7.86	2.05	2.27	0.9
Supervisory support	8.48	1.47	2.47	1.07
Retirement benefit	7.84	1.96	2.43	0.84
Regular pay raises	7.84	1.76	2.58	0.84
Challenge in job	7.83	1.48	3.4	0.98
Overall benefits	7.83	1.8	2.25	0.84
Healthcare benefits	7.37	2.16	2.06	0.93

Using Turnley and Feldman’s (1999) process to calculate the degree of breach of the psychological contract, the degree to which each of the 16 aspects was fulfilled compared to expected was measured on a scale of from − 2 to + 2. For analysis of means, this scale was adapted to a 1–5 Likert scale. Scores of more than 2.5 indicate respondents received as much or more of these items as expected. Scores of less than 2.5 reveal they received less than expected. Results indicate that doctors received more responsibility and job challenge from their employer than they had expected or been promised, with a mean of 3.4 for both (SD 1.05 for responsibility and 0.98 for challenge in job). The lowest scoring item was organizational support, with a mean of 1.98 (SD 0.96) indicating that doctors received less support from the organization than they perceived had been promised to them.

To calculate the degree of psychological contract violation, all responses to the degree of fulfilment were reverse scored so the higher the response, the more significant the breach represented. The weighted degree of breach of the psychological contract was worked out by multiplying the score for the item’s importance by the score for the item’s fulfilment [24]. This weighted degree of breach is equivalent to the level of violation in Turnley and Feldman’s model. The higher the score, the greater the violation, with scores over zero indicating some violation. Scores ranged from − 282 to 210, with an average of 65.4 (SD 67.36). Violation scores can be seen in Table 5.

**Table 5:** Violations of the psychological contract

	Minimum	Maximum	Mean	SD
Organizational support	− 20	20	8.91	8.8
Training	− 20	20	8.49	9.7
Healthcare benefits	− 20	20	7.40	8.01
Feedback	− 20	20	6.38	8.34
Bonuses/overtime payments	− 16	20	6.14	7.7
Overall benefits	− 20	20	6.08	7.39
Retirement benefit	− 20	20	4.77	7.41
Career development	− 20	20	4.70	8.66
Salary	− 20	20	4.69	7.44
Supervisory support	− 20	20	4.68	9.56
Advancement	− 20	20	3.88	8.53
Regular pay raises	− 20	20	3.58	7.32
Job security	− 20	20	3.13	8.209
Decision-making input	− 20	20	1.13	7.48
Challenge in job	− 20	20	− 2.93	7.94
Responsibility	− 20	20	− 3.06	8.71
Total	− 282	210	65.4	67.36

**Table 6:** Hierarchical regression analyses predicting job satisfaction and wellbeing

	Job satisfaction		Flourishing Scale		Affect balance	
	Step 1	Step 2	Step 1	Step 2	Step 1	Step 2
Breach	− 0.667***	− 0.329	− 0.343***	− 0.642**	− 0.378***	− 0.200
Violation		− 0.345		0.306		− 0.182
*F*	223.846***	113.730***	37.1***	19.71***	46.491***	23.418***
∆*F*		2.451		1.209		0.438
∆*R*^2^		0.005		0.004		0.001
Adjusted *R*^2^	0.443***	0.446	0.114***	0.115	0.140***	0.138

*N* = 281. *p < 0.05. **p < 0.01. ***p < 0.001

Violations were most prevalent in breaches of provision of training and organizational support, with means of 8.49 (SD 9.7) and 8.91 (SD 8.8), respectively. Despite a wide range in individuals’ total violation scores, the mean of all respondents is positive 65.4. This indicates that violation of the psychological contract is an issue for non-consultant doctors in Ireland (Table 6).

The ability of psychological contract breach and violation to predict job satisfaction and wellbeing was determined using linear regression analysis, with breach and violation entered in subsequent steps as the independent variables and job satisfaction, the Flourishing Scale and affect balance as the dependent variables. Breaches of the psychological contract led to statistically significant reductions in job satisfaction and wellbeing as measured by the both the Flourishing Scale and affect balance. Violations did not have a significant effect beyond breach as a predictor.

## Discussion

This study has examined the degree of job satisfaction and wellbeing experienced by non-consultant doctors in Ireland, the content of their psychological contract and the extent to which breaches and violations of the psychological contract predict their job satisfaction and wellbeing. Below, we discuss the significance of our findings and place them in the context of the Irish healthcare sector.

### Job satisfaction

Reported levels of overall job satisfaction in this cohort of doctors were reasonably high. Notably, one of the highest scoring individual attributes in the Job Satisfaction Scale was ‘the work you do’. This is a reassuring finding, given that previous research has identified decreases in job satisfaction arising from the changes in doctors’ role from purely clinical to incorporating more administrative duties with correspondingly higher medicolegal risks [7]. The current study, however, suggests that doctors’ day-to-day work is still enjoyed and a positive factor for respondents.

High levels of satisfaction with teamwork and a functioning team dynamic were reported. One benefit of Ireland’s slow implementation of the European Working Time Directive has been that, in many hospitals, a traditionally team-based structure with teams incorporating a consultant, registrar, senior house officer or intern remains in place. This is in contrast to, for example, the UK where the traditional team or ‘firm’-based structure has been eroded due to a shift work pattern and more frequent rotations, as well as the working hours restrictions of the European Working Time Directive. In the UK, there is a push to bring back some elements of the historic team structure as it is now accepted that team work improves job satisfaction for non-consultant doctors [25].

### The psychological contract of non-consultant doctors

The findings of this study make clear that psychological contract breach has a direct, negative association with job satisfaction and wellbeing for doctors, echoing the results of previous research conducted with employees outside the healthcare sector [21]. The current findings give healthcare administrators a potentially significant policy lever with which to improve outcomes for doctors; tackling the mismatch between expectations and reality for the job-related attributes most important to doctors could result in more positive attitudes and experiences of wellbeing, with prospective knock-on effects on retention.

Non-consultant doctors’ expectations may also need to be realigned. Increasing transparency from postgraduate training colleges regarding trainee numbers and expectations from trainees may help this. Career workshops from an early stage with discussions from varying subspecialities may also be beneficial. In 2014 the National Training and Development Programme was launched by the HSE. One of the aims of this programme is to streamline the training of doctors to ensure appropriate workforce planning in the future [26]. This has the potential to be beneficial both for doctors in training and the public at large.

Looking specifically at non-consultant doctors’ perceptions of their psychological contract, there were some notable findings. The three most important areas within the psychological contract to respondents were training, career development and advancement—all areas associated with long-term careers and a relational psychological contract. The most common violations were in organizational support and training. Interestingly, in keeping with this evidence of the importance to doctors of organizational support, the lowest scoring elements of the Job Satisfaction Scale were hospital management and human resources.

With regard to organizational support, this may be evidence of a mismatch in views between employers and employees of the type of psychological contract that exists between them. Non-consultant doctors are usually transient staff, either rotating through their postgraduate placements or on short-term ‘stand-alone’ contracts. Temporary staff usually have a transactional psychological contract with their employer, expecting little in terms of long-term rewards or development [16]. However, despite staying for a relatively short time in each post, it is clear from this study that career progression and training—more relational aspects of a psychological contract [16]—are of utmost importance to non-consultant doctors in Ireland. The disparity may lie here. The disparity may lie here. Despite the temporary contracts with individual hospitals, the doctors see their psychological contract with the health service as a whole and intend to work within that health service over a prolonged period of time—a relational contract. However, in the individual work place these are temporary staff—a transactional contract, in that the hospital administration may see their relationship with a non-consultant doctor as transactional, but the doctor views it as relational. Doctors may not feel the support in their long-term careers from employers and as such feel like they are managing their career progression in isolation, leading to a negative relationship between the doctor and hospital.

With regard to training, despite the formalization of training agreements with postgraduate bodies and hospitals since the Buttimer report [9], the current study reveals that training is still of utmost importance to Irish non-consultant doctors and remains an area where they feel their expectations are not being met. This corresponds with research by Bennett et al. who found that training experiences in Ireland still fall below that available to non-consultant doctors elsewhere in the European Union [27].

### Limitations

There are some limitations to this study. Firstly, as with any online opt-in questionnaire, there is a possible self-selection bias in sampling. People are more likely to complete a survey if they have a strong opinion or problem to voice about the issue at hand, and this may be particularly the case when discussing one’s workplace. This sampling bias may lead to more negative views being expressed than would be the case among the entire population of non-consultant doctors in Ireland. In addition, people are more likely to recall negative experiences and results than positive ones [28]. The results of this study were, however, largely positive for job satisfaction and wellbeing, and scores for these were higher than those found in comparable studies [15]. The large cohort of respondents has likely negated some of this negative bias.

The use of a survey on an open forum also has some limitations. Those non-consultant doctors who do not know about or have access to this forum were automatically excluded. However, it did serve as a means to ensure responses from a diverse group, with respondents from every level of training, varying age ranges and varying years since graduation. Finally, the measures used were not developed solely for healthcare professionals, nor had they been validated on medical staff. Hence, there is potential for unique areas of doctors’ psychological contracts, job satisfaction and wellbeing to have been missed.

## Conclusion

This is the first study to examine the interplay of job satisfaction, wellbeing and the psychological contract of doctors in Ireland. It looks specifically at areas of importance to doctors within their psychological contracts and the extent to which they perceive that these contracts have been breached by their employers. Providing insight into the job-related expectations of non-consultant doctors in Ireland, this study demonstrates a clear mismatch between what doctors expect and what they receive. It identifies specific areas where expectations are not being met, such as organizational support and HR management, and highlights the need for improvements in training and career advancement opportunities. Finally, this study reveals a negative effect of mismatches between expectations and reality on doctors’ job satisfaction and wellbeing. Future research and policy initiatives can build on these findings to explore ways to resolve psychological contract breach in the areas identified here, improving outcomes for doctors and potentially for the healthcare system as a whole.

## Data Availability

The datasets used and/or analysed during the current study are available from the corresponding author on reasonable request.

## Abbreviations

CCST: Certificate of completion of speciality training
SPANE: Scale of positive and negative experiences
SD: Standard deviation
